# DEVELOPMENT, CONTENT VALIDATION, AND USER TESTING OF AN ICU FAMILY WORKBOOK FOR TRANSITIONS FROM HOSPITAL TO HOME

**DOI:** 10.1093/geroni/igae098.2229

**Published:** 2024-12-31

**Authors:** Leslie Scheunemann, Aishwarrya Arivudainambi, Avital Isenberg, Mutasim Makeen, Janelle Christensen, Heidi Donovan

**Affiliations:** University of Pittsburgh, Pittsburgh, Pennsylvania, United States; University of Pittsburgh, Pittsburgh, Pennsylvania, United States; University of Pittsburgh, Pittsburgh, Pennsylvania, United States; University of Pittsburgh, Pittsburgh, Pennsylvania, United States; University of Pittsburgh, Pittsburgh, Pennsylvania, United States; University of Pittsburgh, Pittsburgh, Pennsylvania, United States

## Abstract

**Background:**

No evidence-based interventions provide education and training for families experiencing critical illness to adapt to the caregiving role and attend to self-care while caregiving across transitions from hospital to home.

**Methods:**

We integrated literature with input from community engagement studios to develop a family-facing ICU Family Workbook addressing common unmet needs. A convenience sample of post-ICU family caregivers, clinicians, and researchers qualitatively and quantitatively assessed content validity related to 11 evidence-based elements. Given a strong content validity index, it is undergoing user testing with a convenience sample of 10 families experiencing critical illness to refine its structure and format.

**Results:**

The ICU Family Workbook includes roughly 70 pages of content covering 11 elements: (1) identifying a transitional “system navigator”; (2) education on symptom management; (3) education on functional support; (4) caregiver needs assessment; (5) development of written plans; (6) financial, legal, employment and insurance resources; (7) interdisciplinary decision support; (8) online resources; (9) self-care/psychological support and referral; (10) education on communicating with clinicians; (11) respite resources. Overall content validity index was 0.93. Qualitative feedback informed revisions refining educational content, enhancing screening tools, and making clearer education and self-care recommendations. Early user testing suggests usability and acceptability to families and is informing additional refinement of its content and formatting. Conclusions: This publicly available ICU Family Workbook (www.picturethis.pitt.edu) has content validity for addressing 11 common gaps in caregiver support. After additional refinement based on user testing, it should undergo efficacy testing for improving caregiving and self-care for families after critical illness.

